# A shark's eye view: testing the ‘mistaken identity theory’ behind shark bites on humans

**DOI:** 10.1098/rsif.2021.0533

**Published:** 2021-10-27

**Authors:** Laura A. Ryan, David J. Slip, Lucille Chapuis, Shaun P. Collin, Enrico Gennari, Jan M. Hemmi, Martin J. How, Charlie Huveneers, Victor M. Peddemors, Louise Tosetto, Nathan S. Hart

**Affiliations:** ^1^ Department of Biological Sciences, Macquarie University, North Ryde, New South Wales 2109, Australia; ^2^ Taronga Conservation Society Australia, Bradley's Head Road, Mosman, New South Wales 2088, Australia; ^3^ Biosciences, College of Life and Environmental Sciences, University of Exeter, Exeter EX4 4QD, UK; ^4^ School of Life Sciences, La Trobe University, Bundoora, Victoria 3086, Australia; ^5^ Oceans Research Institute, Mossel Bay 6500, South Africa; ^6^ South African Institute for Aquatic Biodiversity, Private Bag 1015, Grahamstown 6140, South Africa; ^7^ Department of Ichthyology and Fisheries Science, Rhodes University, Grahamstown 6140, South Africa; ^8^ School of Biological Sciences and The UWA Oceans Institute, M092, University of Western Australia, Perth, Western Australia 6009, Australia; ^9^ School of Biological Sciences, University of Bristol, Bristol BS8 1TQ, UK; ^10^ College of Science and Engineering, Flinders University, Bedford Park, South Australia 5042, Australia; ^11^ New South Wales Department of Primary Industries, Sydney Institute of Marine Science, Mosman, New South Wales 2088, Australia

**Keywords:** shark attack, prey detection, shape discrimination, object motion, shark vision

## Abstract

Shark bites on humans are rare but are sufficiently frequent to generate substantial public concern, which typically leads to measures to reduce their frequency. Unfortunately, we understand little about why sharks bite humans. One theory for bites occurring at the surface, e.g. on surfers, is that of mistaken identity, whereby sharks mistake humans for their typical prey (pinnipeds in the case of white sharks). This study tests the mistaken identity theory by comparing video footage of pinnipeds, humans swimming and humans paddling surfboards, from the perspective of a white shark viewing these objects from below. Videos were processed to reflect how a shark's retina would detect the visual motion and shape cues. Motion cues of humans swimming, humans paddling surfboards and pinnipeds swimming did not differ significantly. The shape of paddled surfboards and human swimmers was also similar to that of pinnipeds with their flippers abducted. The difference in shape between pinnipeds with abducted versus adducted flippers was bigger than between pinnipeds with flippers abducted and surfboards or human swimmers. From the perspective of a white shark, therefore, neither visual motion nor shape cues allow an unequivocal visual distinction between pinnipeds and humans, supporting the mistaken identity theory behind some bites.

## 1. Background

Although shark bites on humans are rare, they can have a devastating effect on victims and first responders and negative economic impacts on local communities [1,2]. Bites also have negative consequences for sharks as they often result in the implementation or continued use of lethal shark mitigation measures, including the deployment of gill nets and drum lines to reduce shark populations [3,4]. Such measures can impact vulnerable shark populations and affect non-target species caught unintentionally as bycatch [5,6]. Public support for these invasive mitigation measures is thought, at least in part, to reflect the disproportionate level of fear associated with sharks [7,8] and the uncertainties that still surround the reasons for, and possible prevention of, shark bites [9,10].

Numerous shark species are known to have bitten humans, but three species are responsible for most injuries and fatal bites, white sharks (*Carcharodon carcharias*), bull sharks (*Carcharhinus leucas*) and tiger sharks (*Galeocerdo cuvier*) [11–13]. Why sharks sometimes bite humans remains unclear, but potential reasons include mistaken identity, whereby sharks are thought to mistake humans for their typical prey; curiosity; hunger; and defensive/offensive aggression [14,15]. Shark bites are often categorized as either ‘provoked’ or ‘unprovoked’. A provoked shark bite may be an aggressive/defensive behaviour as a result of a direct disturbance by a human, such as a diver touching a shark, a fisher catching or spearing a fish or shark [16] or intrusion into a shark's territory [17,18]. Unprovoked bites are the most puzzling and arguably generate the most fear.

Predatory behaviour has been studied extensively in white sharks, which are thought to rely heavily on vision to detect and target their prey, especially at close range (up to approx. 15 m) [19]. White sharks are more successful when hunting prey located at the surface, where the silhouette probably aids the identification of prey against the background skylight [20–22]. In addition, white sharks have visual adaptations that enhance prey detection at the surface, such as cone photoreceptors and a retinal region for acute vision (area centralis) that samples the dorso-lateral region of the visual field, a zone above and to the sides of the head [23].

As a group, surfers are at the highest risk of fatal shark bites, particularly from juvenile white sharks [11,13,24]. The potential similarity in visual appearance of surfers and pinnipeds when viewed by white sharks from below has long been postulated as a potential cause of shark bites [14]. Indeed, white sharks appear to elicit a similar prey capture behaviour towards pinnipeds and humans, which suggests that some bites may result from mistaken identity [14,25]. Following an initial strike on a pinniped, white sharks typically retreat, allowing the animal to weaken and bleed extensively before returning to feed [14,25]. Humans are also usually released after the initial strike, although the shark rarely returns to consume the victim [26,27]. This behavioural difference may be partly attributed to the removal of a shark bite victim from the water before the shark can consume them, intervention from other people or the victim fighting back. However, it may also suggest that white sharks do not actively seek out humans as prey and that bites may be a case of mistaken identity.

On the other hand, indirect evidence, based on implied bite force, suggests that most bites on humans are caused by juvenile white sharks and that they can discriminate humans from pinnipeds [24]. Forensic comparison of bites on pinnipeds and humans suggests that white sharks use greater bite force when attacking pinnipeds, which could indicate that bites on humans are more exploratory or tempered, and are not simply the result of mistaken identity [24]. However, it is also possible that similarities in the visual, auditory and/or hydrodynamic cues emitted by humans and pinnipeds might initially trigger a bite and that only at closer range do differences in electromagnetic, gustatory and/or proprioceptive cues cause white sharks to reduce the intensity of their bite. Factors such as water depth, approach angle and intraspecific variation in behaviour may also cause differences in the severity of bites on pinnipeds and humans.

The mistaken identity theory has received little scientific scrutiny and the visual similarity between humans and pinnipeds at the surface has been debated largely on the basis of human visual perception, rather than that of sharks [14]. However, recent progress in our understanding of the shark visual system enables us to investigate further the similarities between pinnipeds and humans from a shark's perspective. Sharks are completely colour blind or at best have only limited colour perception [28,29]. Sharks also have poor spatial resolving power, with the highest estimates based on retinal anatomy at approximately 10 cycles per degree (cpd; range 2–10 cpd) [30], which is considerably worse than humans (30 cpd) [31]. Benthopelagic and pelagic species that feed on more mobile prey have higher spatial resolving power [31,32]. Temporal resolution and contrast sensitivity have been measured in a few elasmobranchs. Temporal resolution is higher in species from brighter light environments (range 12–44 Hz) and contrast sensitivity does not vary significantly between the benthic species it has been measured in, all detecting contrasts below 2.5% [33–36]. Taken together, these findings suggest that motion and brightness contrast are likely to be the primary visual cues used by most sharks to detect and target prey [33,34]. What is still required, however, is the interrogation of the visual cues emitted by relevant prey items and humans in the water, as perceived by sharks.

In this study, we measured and compared the visual cues emitted by different objects from the perspective of juvenile white sharks to test the mistaken identity hypothesis. Video footage of pinnipeds swimming, humans swimming, humans paddling surfboards and a moving rectangular float was obtained from the perspective of a shark viewing the objects from below, silhouetted against the surface. The videos were filtered digitally using spatial and temporal parameters derived from or estimated for the visual system of juvenile white sharks to quantify the visual motion and shape cues of the objects at the level of the retina. Motion cues were analysed using a two-dimensional motion detection (2DMD) model and shape was analysed based on the distance between the object's centroid and the perceived edges of the object. We hypothesized that the visual motion cues and shape characteristics of human swimmers and surfers would be indistinguishable from those of pinnipeds.

## 2. Material and methods

### 2.1. Study site and animals

Video recordings of the pinnipeds, humans, surfers and rectangular floats were made in the aquarium facilities at Taronga Zoo, Sydney, Australia, to assess their visual similarity. Video recordings were made of two Australian sea lions (*Neophoca cinerea*; mass = 48 kg and 180 kg) and one New Zealand fur seal (*Arctocephalus forsteri*; mass = 48 kg). Two humans were recorded swimming different strokes, including ‘dog paddle’, in which the hands remained in the water, slow freestyle and fast freestyle. The visual cues of three differently shaped surfboards were also assessed: a standard shortboard (1.77 × 0.50 m), a longboard (2.83 × 0.58 m) and a hybrid board, which is similar in shape to the longboard but smaller, with a similar size to the shortboard (1.77 × 0.51 m). Surfboards were paddled at a variety of speeds, both with and without kicking of the legs. For comparison, video footage of a white 0.8 × 0.5 m rectangular float, made from polypropylene foam, towed at the surface was also obtained. The float was attached via a rope to a swimmer and towed directly over the camera. Video recordings of all objects were made in two aquaria (large: depth = 4.5 m; small: depth = 3.3 m). Footage of pinnipeds was only recorded in the aquarium in which they were housed.

The human and pinniped comparison was performed using both a stationary and a mobile camera rig. The two rigs allowed the visual cues of the objects to be assessed while accounting for the predicted self-motion of sharks, as well as from a stationary perspective. The stationary perspective was important to isolate object motion and remove random variation experienced in the moving perspective. The stationary footage was recorded from a GoPro Hero 3 camera (resolution 1920 × 1080 pixels, frame rate 30 frames per second (fps)) weighted down on the bottom of the aquarium, with cameras facing the surface. The mobile unit comprised a GoPro Hero 3 camera mounted on a Seadoo GTS underwater scooter. The scooter has a top speed of 1.25 m s^−1^, which is comparable to the cruising speed of many large predatory sharks [37]. The scooter was steered along a 10 m transect at constant depth on the bottom of the aquarium, with cameras facing the surface.

### 2.2. Motion analysis

For the stationary video experiment, three video clips (approx. 1–4 s long) of each object, in each aquarium, were used in the analysis, which was performed using custom scripts written in Matlab (R2015; MathWorks). For the mobile experiments, four clips of each object, in each aquarium, were analysed: in two of the clips the scooter and object moved in the same direction and in the other two clips the scooter and object moved in opposite directions. All videos were rotated so that the objects moved from the bottom to the top of the screen. As most sharks are thought to be cone monochromats, including white sharks ([38], N Hart 2021, unpublished data), with their spectral sensitivity peaking in the medium wavelength (green) part of the visible spectrum, only the green channel of the colour RGB video file was used to provide achromatic information.

Motion analysis was performed on a 2.7 × 2.3 m region of interest (ROI) at the surface of the water in the static experiments and a 3 × 1.8 m ROI for the mobile experiments. The analysis started when half of the object entered the ROI and was stopped when half of the object left the ROI. The object was tracked as it moved through the ROI, so that only motion in a rectangular bounding box, the size of the object plus a 20-pixel buffer, was compared. To compare motion cues of objects at different positions on the objects, we also divided the ROI, from the centre to the corners, into four different ‘faces’, i.e. to compare the leading edge, trailing edge and left and right sides between objects.

The visual motion cues of the objects were compared by analysing the videos with a 2DMD model [39,40]. The 2DMD model uses two orthogonal arrays of elementary motion detectors to compare each pixel at a given pixel spacing and between frames based on a given temporal filter. Owing to the large size and protected status of white sharks, it was not possible to measure its temporal resolution using behavioural or physiological experiments. However, the frame rate of the videos (30 fps, i.e. 30 Hz) is similar to the temporal resolution thresholds of carcharhiniform sharks inhabiting similar light environments to white sharks (19–31 Hz) [34–36]. We, therefore, adopted a temporal filter of 30 Hz in the model.

A spacing parameter of 5 cpd was used, based on estimates of the maximum anatomical spatial resolving power obtained from analysis of the retinas of two juvenile white sharks (electronic supplementary material, figure S1). Previous studies in elasmobranchs indicate that, in most cases, behavioural and electrophysiological estimates of spatial resolving power are substantially lower than anatomical estimates [33,34,41–43]. Thus, we also modelled motion cues using a spacing parameter of 2.5 cpd, which more closely reflects the spatial resolving power across the majority of the retina (i.e. outside the area centralis) and also correlates with the known receptive field size of ganglion cells measured in other elasmobranchs [42,43].

Each video clip was analysed to determine the strength of motion for 72 vectors relative to the direction travelled, with each vector being the unweighted mean of the five 1° vectors within a 5° arc. The motion strength for each direction/vector was compared between objects using mixed models in R (v. 1.1.143, RStudio, Inc., Boston, MA, USA) based on the *lme4* package [44]. Mixed models were also used to assess total motion and motion strength at the four different ‘faces’ of the objects (face 1 = leading edge, face 2 = left side, face 3 = trailing edge and face 4 = right side). Each face was defined as the portion of the object outline that fell within each quadrant defined by diagonal lines crossing the ROI.

Motion strength was log_10_ transformed to fulfil the assumption of homogeneity of variance [45]. The identification of the video clip and the aquarium used was treated as a random factor, and models were compared using the ANOVA function in R. Models were also assessed based on Akaike's information criterion (AIC). The strengths of motion cues were normalized within each aquarium by dividing each vector motion strength by the largest motion recorded in a single motion vector within each aquarium. Normalization was performed to account for uncontrollable factors such as water visibility and light environment, which varied between aquaria and filming sessions. For the mobile video analysis, the direction the object was moving relative to the camera was also included as a random factor. After establishing an overall difference between objects, pairwise comparisons were performed for each direction vector using the *lsmeans* package [46] to determine which direction vectors drive the overall result.

### 2.3. Shape analysis

The static video footage was also used in the shape analysis. Nine image frames from the video footage were selected randomly to compare the shape of (i) the three individual pinnipeds in a streamline position with both flippers adducted; (ii) the same pinnipeds with both flippers abducted; (iii) the two individual swimmers; (iv) the standard shortboard surfboard; (v) the longboard surfboard; and (vi) the hybrid surfboard. The longboard was only analysed in five frames from the large aquarium because, when placed in the small aquarium, the longboard was too large to fit in a single video frame.

All image frames were processed to reflect the visual abilities of juvenile white sharks using a custom Matlab code. The edges of the objects were detected using the same Gaussian filter used in the motion analysis. The edges were then used to fill the object to create a binary image. Two methods were used to assess shape: (i) roundness—where the centroid of the object was calculated and the mean distance from the centroid to the edge calculated every 2°; and (ii) edge projection—where the perimeter of the object was divided into 180 curves of equal length and the mean distance from the centroid to each curve calculated. A fast Fourier transformation (FFT) was then performed on the edge distances. To eliminate apparent differences in shape due to object size alone, a normalized FFT was also performed [47,48]. Mixed models were used to compare the FFT amplitudes of the first 20 frequencies using the *lme4* package [44], and model terms were compared using ANOVA. Frequency was treated as a categorical variable and each image frame was assigned a unique identification number that was set as a random factor. FFT amplitude was log_10_ transformed. To determine how shape differed between objects, a pairwise comparison was performed for each FFT frequency using the *lsmeans* package [46].

## 3. Results

### 3.1. Motion analysis—static footage

**Figure 1.**
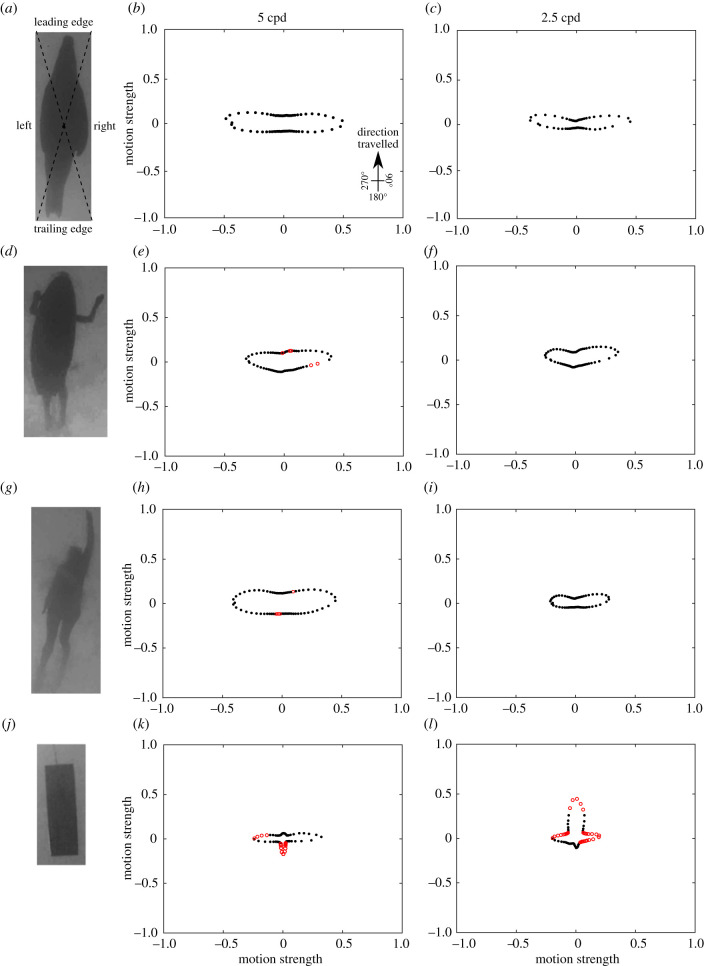
Direction and strength of motion cues from the 2DMD model of (*a*–*c*) a pinniped swimming, (*d*–*f*) a human paddling a surfboard, (*g*–*i*) a human swimming and (*j*–*l*) a rectangular float towed through the water. Modelling was performed assuming a spatial resolving power of either 5 cpd (*b*,*e*,*h*,*k*) or 2.5 cpd (*c*,*f*,*i*,*l*). Red open dots indicate angle vectors that were significantly different from the pinniped and black dots were not significantly different. Units are an arbitrary scaling value. Dashed lines on (*a*) show the division of the object for analysis of the different ‘faces’. Only the rectangular float differed from the pinniped at 2.5 cpd.

The humans swimming, surfboards being paddled, towed rectangular float and pinnipeds swimming at the surface of the water, at a spatial resolving power of 5 cpd and 2.5 cpd, varied in motion magnitude at different angle vectors (table 1 and figure 1). At 5 cpd, the swimmer and surfer differed significantly from the pinniped at five out of 72 vectors, whereas the rectangle differed at 14 vectors. The rectangle differed from the pinniped and other objects as it produced greater visual motion in the direction opposing the direction travelled. The motion cues emitted by the pinnipeds, surfboards being paddled and swimmers were strongest in the sidewards directions, perpendicular to the direction travelled (figure 1). At 2.5 cpd, only the rectangle differed from the pinniped, and they differed at 33 motion vectors. The rectangle produced greater visual motion in the direction travelled and less visual motion in directions diagonal to the direction travelled (figure 1).

**Table 1.** Mixed model results showing the significant difference between objects at 5 cpd and 2.5 cpd when the whole object was analysed and each of the faces (face 1 = leading edge, face 2 = left side, face 3 = trailing edge and face 4 = right side). Degrees of freedom equals 216. * *p* < 0.05.

face	resolution (cpd)	model	AIC	*χ*^2^	*p*-value
all	5	vector	−2924		
		vector × object	−3628	1136.30	<0.001*
	2.5	vector	−2157		
		vector × object	−4270	2545.3	<0.001*
face 1	5	vector	−5820		
		vector × object	−6233	844.65	<0.001*
	2.5	vector	−4078		
		vector × object	−5434	1782.1	<0.001*
face 2	5	vector	−1865		
		vector × object	−2219	786.36	<0.001*
	2.5	vector	−1865		
		vector × object	−2319	876.44	<0.001*
face 3	5	vector	−2013		
		vector × object	−3084	1502.5	<0.001*
	2.5	vector	−1115		
		vector × object	−2980	2297.1	<0.001*
face 4	5	vector	−2516		
		vector × object	−2648	563.4	<0.001*
	2.5	vector	−2499		
		vector × object	−2801	734.5	<0.001*

**Figure 2.**
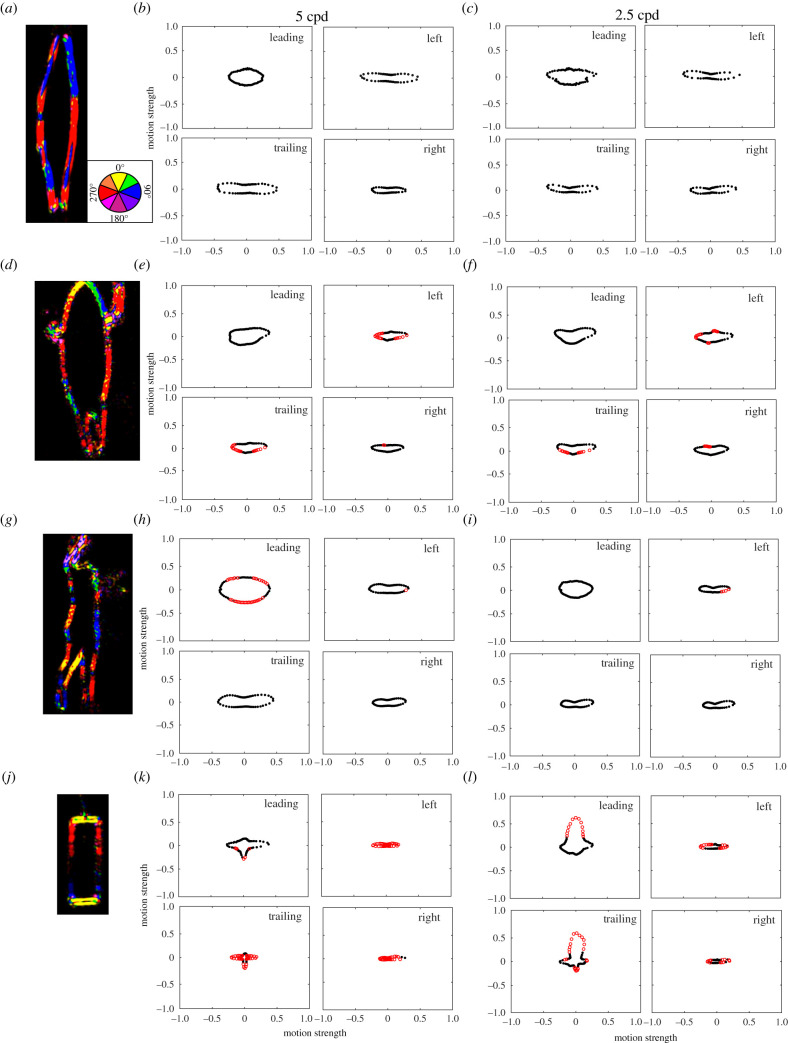
Mean direction and strength of motion cues for each object from the 2DMD model of (*a*–*c*) a pinniped swimming, (*d*–*f*) a human paddling a surfboard, (*g*–*i*) a human swimming and (*j*–*l*) a rectangular float towed through the water, calculated for the four different faces (leading edge, left side, trailing edge, right side) at 5 cpd (*b*,*e*,*h*,*k*) and 2.5 cpd (*c*,*f*,*i*,*l*). Red open dots represent motion directions that were significantly different from that of the pinniped and black dots were not significantly different. Units are an arbitrary scaling value. Panels (*a*,*d*,*g*,*j*) show example frames of motion direction over pixel location, where pixel colour corresponds to the motion direction in the colour wheel (inlay *a*).

Comparison of the objects by face 1 (leading edges) also found that objects varied in the motion magnitude at different angle vectors at both 5 cpd and 2.5 cpd (table 1). At 5 cpd, the surfer did not vary from the pinniped, the swimmer varied from the pinniped at 35 motion vectors out of 72 vectors and the rectangular float differed at five angle vectors. The shape of motion cues, when plotted as a function of motion direction, was similar between the swimmer and pinniped, although the swimmer created greater motion cues in all directions (figure 2). The rectangle produced greater motion opposing the direction travelled. At 2.5 cpd, the surfer and swimmer did not vary from the pinniped, whereas the rectangular float differed at 13 angle vectors. The rectangle produced greater motion in the direction travelled (figure 2).

Comparison of the objects by face 2 (left side) also found that objects varied in the motion magnitude at different angle vectors (table 1). At 5 cpd, the surfer varied from the pinniped at 17 motion vectors out of 72 vectors, the swimmer varied from the pinniped at one motion vector and the rectangular float differed at all angle vectors. The shape of motion cues, when plotted as a function of motion direction, was similar between all objects; however, the surfer created weaker motion cues perpendicular to the direction travelled (figure 2). The rectangle produced weaker motion in all directions (figure 2). At 2.5 cpd, the surfer varied from the pinniped at 15 motion vectors, the swimmer varied from the pinniped at four motion vectors and the rectangular float differed at 18 angle vectors. The shape of motion cues, when plotted as a function of motion direction, was still similar between all objects; however, the surfer created weaker motion cues perpendicular to the direction travelled and stronger cues in the direction travelled and the opposing direction. The swimmer and rectangle produced weaker motion perpendicular to the direction travelled (figure 2).

Comparison of the objects by face 3 (trailing edge) also found that objects varied in the motion magnitude at different angle vectors (table 1). At 5 cpd, the surfer varied from the pinniped at 19 motion vectors out of 72 vectors, the swimmer did not vary from the pinniped and the rectangular float differed at 46 angle vectors. The shape of motion cues, when plotted as a function of motion direction, was similar between the surfer and pinniped; however, the surfer created weaker motion cues perpendicular to the direction travelled (figure 2). The rectangular float produced greater motion opposing the direction travelled and less motion diagonal to the direction travelled. At 2.5 cpd, the surfer varied from the pinniped at 13 motion vectors, the swimmer did not vary from the pinniped and the rectangular float differed at 21 angle vectors. The shape of motion cues, when plotted as a function of motion direction, was similar between the surfer and pinniped; however, the surfer produced weaker motion cues at angles approximately 135° to the direction travelled. The rectangle produced much greater motion in the direction travelled and the opposing direction (figure 2).

Comparison of the objects by face 4 (right side) also found that objects varied in the motion magnitude at different angle vectors (table 1). At 5 cpd, the surfer varied from the pinniped at two motion vectors out of 72 vectors, the swimmer did not vary from the pinniped and the rectangular float differed at 70 angle vectors. The rectangular float differed from the pinniped and other objects as it produced weaker motion in all directions (figure 2). At 2.5 cpd, the surfer varied from the pinniped at six motion vectors, the swimmer did not vary from the pinniped and the rectangular float differed at 13 angle vectors. The shape of motion cues, when plotted as a function of motion direction, was similar between all objects; however, the surfer produced stronger motion in the direction travelled. The rectangle produced much weaker motion perpendicular to the direction travelled (figure 2).

### 3.2. Motion analysis—mobile footage analysis

We analysed the mobile footage to compare motion strength as a function of vector direction between pinnipeds, surfers and swimmers. At 2.5 cpd, there was a significant difference in motion magnitude between the objects at different angle vectors (vector, AIC = −1833; vector × object, AIC = −2983; mixed model, $\chi_{144}^{2}=1437.7$, *p* < 0.001*). The pinniped varied from the surfer at 50 motion vectors and the swimmer at 53 motion vectors. The objects had a similar angular distribution of motion cues, with most motion produced perpendicular to the direction travelled. However, both the surfer and swimmer produced greater motion in the direction travelled and the opposing direction (electronic supplementary material, figure S2). In all ‘faces’ there was greater motion in the direction travelled and the opposing direction for the human paddling a surfboard and the human swimming in comparison with pinnipeds (electronic supplementary material, table T1 and figure S3).

### 3.3. Shape

**Figure 3.**
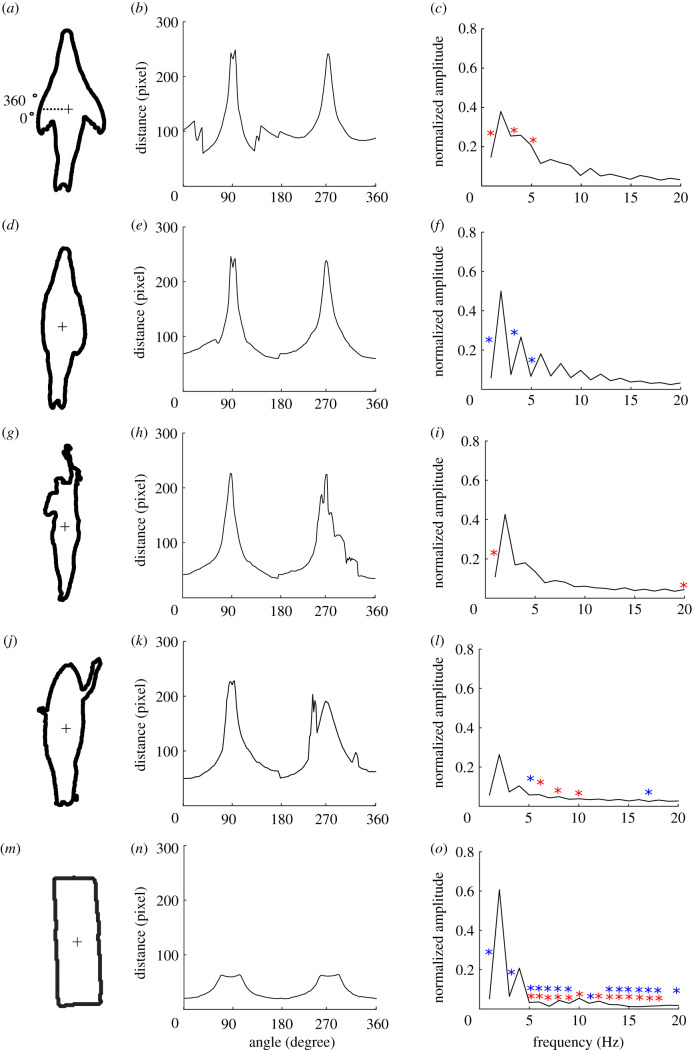
Five examples of the roundness shape analysis for the different object categories. (*a*–*c*) A pinniped with its flippers abducted; (*d*–*f*) a pinniped in a streamlined position; (*g*–*i*) a human swimming; (*j*–*l*) a human paddling a surfboard; and (*m*–*o*) a rectangular float. The mean distance from the centroid to the edge of the shape measured in 2° increments (*b*,*e*,*h*,*k*,*n*) and the normalized FFT amplitudes (*c*,*f*,*i*,*l*,*o*) are also shown. Red asterisks represent FFT frequencies that were significantly different from the pinniped in a streamlined pose, and blue asterisks represent FFT frequencies that were significantly different from the pinniped with its flippers abducted.

The roundness analysis found the normalized FFT amplitude was significantly different at a number of Fourier descriptors between the different objects (Fourier descriptor + object, AIC = 282; Fourier descriptor × object, AIC = 119; mixed model, $\chi_{80}^{2}=323.0$, *p* < 0.001). There was little difference, however, between the shape of the swimmers, the shortboard surfboard and the pinnipeds with their flippers in the abducted position, where no Fourier descriptor was significantly different between the swimmer and pinniped with flippers abducted and only two frequencies differed between the surfer and pinniped with flippers abducted (figure 3). Three Fourier descriptors were significantly different between the pinnipeds in a streamline position compared with when its flippers were in the abducted position.

**Figure 4.**
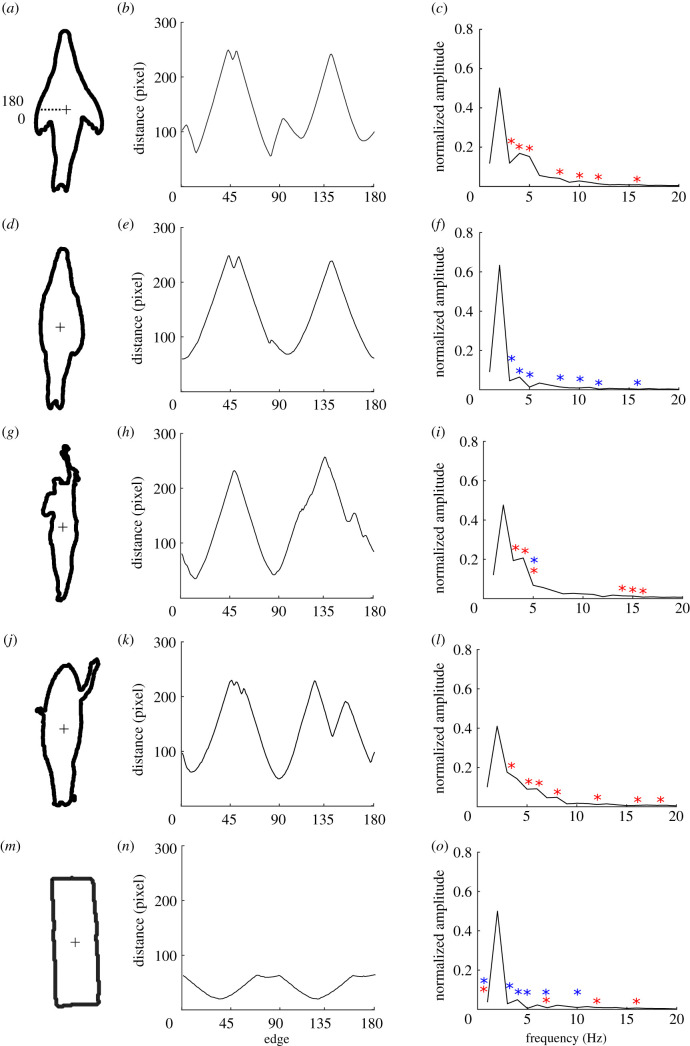
Five examples of the edge projection shape analysis for the different object categories. (*a*–*c*) A pinniped with its flippers abducted; (*d*–*f*) a pinniped in a streamlined position; (*g*–*i*) a human swimming; (*j*–*l*) a human paddling a surfboard; and (*m*–*o*) a rectangular float. The mean distance from the centroid to the edge, divided into 180 equal length curves (*b*,*e*,*h*,*k*,*n*), and the normalized FFT amplitudes (*c*,*f*,*i*,*l*,*o*) are also shown. Red asterisks represent FFT frequencies significantly different from the pinniped in a streamline position and blue asterisks represent FFT frequencies significantly different from the pinniped with its flippers abducted.

The edge projection analysis found that the normalized FFT amplitude was significantly different at a number of Fourier descriptors between the different objects (Fourier descriptor + object, AIC = 466; Fourier descriptor × object, AIC = 281; mixed model, $\chi_{80}^{2}=345$, *p* < 0.001). There was little difference, however, between the shape of the swimmers, the shortboard surfboard being paddled and the pinnipeds with their flippers in the abducted position, where only one Fourier descriptor was different between the swimmer and pinniped with flippers abducted and no frequencies differed between the surfer and pinniped with flippers abducted (figure 4). Seven Fourier descriptors were significantly different between the pinnipeds in a streamline position compared with when its flippers were in the abducted position. Both the roundness and edge projection analysis shows that a pinniped swimming with its flippers abducted is more similar to a human swimmer and a shortboard surfboard being paddled than it is to a pinniped in a streamline position. The rectangular float was more similar to the pinniped in a streamline position but still differed at four Fourier descriptors.

The three different-shaped surfboards were also compared with shapes of the pinnipeds in a streamline position with flippers abducted. Both the roundness and edge projection analyses showed that the normalized FFT amplitude was significantly different at several Fourier descriptors between the different objects (roundness: Fourier descriptor + object, AIC = 247; Fourier descriptor × object, AIC = 237; mixed model, $\chi_{80}^{2}=170.3$, *p* < 0.001, edge: Fourier descriptor + object, AIC = 183; Fourier descriptor × object, AIC = 150; mixed model, $\chi_{80}^{2}=192.7$, *p* < 0.001). Both the longboard and the hybrid surfboard were also more different from the pinniped than the shortboard surfboard (electronic supplementary material, figure S4). Thus, the shortboard surfboard was considered more similar than the other shaped surfboards to the pinnipeds and used in the comparison with the swimmer and the rectangular float.

## 4. Discussion

### 4.1. Motion cues

From the visual perspective of juvenile white sharks, the visual motion cues of both humans swimming and paddling a surfboard were not significantly different from pinnipeds swimming at the surface with a spatial resolving power of 2.5 cpd, and there was little difference at 5 cpd. The motion cues generated by these objects were strongest in the sideways directions, perpendicular to the direction travelled. The similarity in the distribution of motion vectors between these objects is due to the tapered leading and/or trailing edges of the objects. By contrast, the straight leading and trailing edges of the rectangle causes most motion to occur in the direction travelled and opposing the direction travelled. Importantly, these estimated motion cues represent first-order motion detection, such as that generated by direction-selective ganglion cells in the retina, and higher order processing of motion is likely to allow sharks to determine the overall direction in which an object is travelling, as is the case in many other vertebrates that have second-order (i.e. texture-contrast modulations) and third-order (tracking features) motion systems [49,50]. However, these first-order motion features may result in uncertainty during object detection or recognition.

We suggest that the analysis performed at 2.5 cpd might better reflect the comparison of objects from the perspective of juvenile white sharks. The main difference between the analyses conducted at 5 cpd and 2.5 cpd is that at 5 cpd more of the water movement around the objects was detected as motion by the 2DMD model. These experiments were conducted in aquaria partly because they provided controlled environments in which to film the objects; however, water visibility in the aquaria was greater than typically found in coastal waters where most shark bites occur—where absorption and scattering of light by suspended particles would reduce visual contrast and effectively filter out high spatial frequencies before they reach the eye. In this study, we did not account for the reduction in visual contrast due to the properties of water. Therefore, our modelling probably overestimates the motion cues available, and the different objects are likely to appear more similar than suggested here, even at 5 cpd. Moreover, in other sharks, both behavioural and electrophysiological estimates of spatial resolving power are substantially lower than anatomical estimates [33,34,41–43]. Thus, while 5 cpd is potentially the maximum resolving power of juvenile white sharks, the resolving power may be less. Modelling with a spatial resolving power of 2.5 cpd would also reflect a scenario where a shark detects an object from a greater distance (approx. 9 m) than that used here, which may apply to a white shark that first identifies pinniped prey from below, at distances greater than 10 m, depending on water clarity [51,52].

Although some differences were detected, particularly in the mobile footage, the motion cues of humans paddling surfboards, humans swimming and pinnipeds were similar in shape and differences in the overall strength of motion arose as a result of the speed and degree of streamlining of the objects. The pinnipeds were faster, more streamlined and required fewer arm strokes than the swimmer and human paddling the surfboard.

### 4.2. Shape

Pinnipeds with their flippers abducted were more similar to the shortboard surfboard and swimmers than to streamlined pinnipeds with their flippers adducted. The fourth Fourier descriptor, which depicts a small protrusion (such as the arms of a surfer and swimmer or pinniped flippers), was the defining similarity between the human paddling the surfboard, the swimmer and the pinniped with its flippers abducted. All objects were similar in roundness and edge projection at the second Fourier descriptor, which describes an object that has a greater length than its width; even the rectangular float had some similarities to the pinnipeds in this regard. Interestingly, white sharks are known to bite rectangular floats [21] and so aspect ratio alone may be an important visual cue of any silhouette. Nevertheless, the rectangle was easily distinguished in the roundness analysis owing to its broader leading and trailing edge.

The shapes of the longboard and hybrid surfboard were less similar than the shortboard surfboard was to that of the pinnipeds, mainly because of its roundness (electronic supplementary material, figure S5), as both the longboard and hybrid board have a broader ‘nose’. There is evidence of longboards, kayaks and stand-up paddleboards being bitten by sharks [13]; however, our modelling would suggest that there may be greater risks associated with smaller objects that more closely resemble the shape of pinnipeds. Prey selection based on size is thought to occur in white sharks, as they specifically target smaller, young pinniped pups [20]. Moreover, there is some evidence that white sharks discriminate based on visual cues when faced with a choice, selecting for the most visually relevant object [21]. However, it is unknown whether, if presented with a choice, white sharks would selectively target a shortboard surfboard over a longboard surfboard.

### 4.3. Validity of the mistaken identity theory

We found that the putative first-order visual motion and shape cues of a human either swimming or paddling a surfboard were statistically non-discriminable from those of a pinniped when analysed using a ‘virtual’ shark visual system. This study provides the first evidence in agreement with the ‘mistaken identity theory’, in which white sharks bite humans because of their visual similarity to their natural pinniped prey. The motion and shape analysis was tailored specifically to juvenile white sharks as they are responsible for the majority of human fatalities [11–13] and pinnipeds are a common prey item [53,54]. However, white sharks do not exclusively feed on pinnipeds and are opportunistic foragers with broad dietary niches [53,54]. Thus, white sharks may associate a broader range of both motion and shape visual cues as potential prey.

The spatial parameters of the motion and shape analysis were based on data from juvenile white sharks. Spatial resolving power may change with age such that adult sharks will have greater spatial resolving power, primarily because of their larger eyes and a correspondingly longer focal length [55]. Therefore, more visual features of the pinnipeds may be distinguishable from those of humans as they age. We were unable to obtain suitable retinal material from larger adult white sharks to assess this possibility. Nevertheless, juvenile sharks are most relevant for the mistaken identity theory because sharks of 2.5–3.5 m total length are responsible for a large proportion of bites on humans [24], which is believed to be linked to juvenile white sharks beginning to incorporate pinnipeds in their diet [53,54].

This study supports the mistaken identity theory from a visual perspective, but sharks also receive information through their other sensory systems, including electroreception, olfaction, audition and the mechanosensory lateral line. For example, white sharks have relatively large olfactory bulbs, suggesting that olfactory cues may be important in predation [56]. Thus, it may be possible for white sharks to discriminate humans from pinnipeds based on other sensory cues. However, there is evidence to suggest that visual cues alone are sufficient to trigger a predatory or exploratory approach; white sharks are known to attack pinniped-shaped decoys and even inanimate objects such as seaweed and rubbish floating at the surface, which do not emit olfactory and/or electrical cues resembling pinnipeds or other prey [21,57]. Moreover, rather than aiding discrimination, other sensory cues such as vibration and sound may in fact enhance the appearance of an object as potential prey. While it seems unlikely that every bite on a human by white sharks is a result of mistaken identity, our results suggest that in circumstances where surface objects, like surfers, are targeted by white sharks from below it is very possible.

The mistaken identity theory may also apply to other species of sharks responsible for human fatalities, such as *G. cuvier* and *C. leucas*. Both species have broad dietary niches and consume large prey items such as turtles [58,59], for which humans could potentially be mistaken. Detritus and surface-dwelling animals (i.e. birds) have been found in the stomach contents of both species, suggesting that they also bite and/or consume potential prey at the surface [58,59].

In conclusion, our results indicate that the poor spatial resolving power of the shark retina may result in bites on humans as a result of mistaken identity or ambiguous visual cues. Modelling here was done under ideal viewing conditions, so this scenario is likely to be of greater significance under more realistic conditions of dim light, surface chop or turbid water.

## Data accessibility

The data are provided in the electronic supplementary material [60].

## References

[1] Hazin FHV, Burgess GH, Carvalho FC. 2008 A shark attack outbreak off Recife, Pernambuco, Brazil: 1992–2006. Bull. Mar. Sci. **82**, 199-212.

[2] Simmons P, Mehmet MI. 2018 Shark management strategy policy considerations: community preferences, reasoning and speculations. Mar. Policy **96**, 111-119. (10.1016/j.marpol.2018.08.010)

[3] Dudley S, Cliff G. 2010 Shark control: methods, efficacy, and ecological impact. In Sharks and their relatives II: biodiversity, adaptive physiology and conservation (eds JC Carrier, JA Musick, MR Heithaus), pp. 567-592. Boca Raton, FL: CRC Press.

[4] McPhee DP, Blount C, Smith MPL, Peddemors VM. 2021 A comparison of alternative systems to catch and kill for mitigating unprovoked shark bite on bathers or surfers at ocean beaches. Ocean Coast. Manag. **201**, 105492. (10.1016/j.ocecoaman.2020.105492)

[5] Reid DD, Robbins WD, Peddemors VM. 2011 Decadal trends in shark catches and effort from the New South Wales, Australia, Shark Meshing Program 1950–2010. Mar. Freshw. Res. **62**, 676-693. (10.1071/MF10162)

[6] Broadhurst MK, Cullis BR. 2020 Mitigating the discard mortality of non-target, threatened elasmobranchs in bather-protection gillnets. Fish. Res. **222**, 105435. (10.1016/j.fishres.2019.105435)

[7] Simmons P, Mehmet M, Curley B, Ivory N, Callaghan K, Wolfenden K, Xie G. 2021 A scenario study of the acceptability to ocean users of more and less invasive management after shark-human interactions. Mar. Policy **129**, 104558. (10.1016/j.marpol.2021.104558)

[8] Crossley R, Collins CM, Sutton SG, Huveneers C. 2014 Public perception and understanding of shark attack mitigation measures in Australia. Hum. Dimens. Wildl. **19**, 154-165. (10.1080/10871209.2014.844289)

[9] Neff C. 2012 Australian beach safety and the politics of shark attacks. Coast. Manag. **40**, 88-106. (10.1080/08920753.2011.639867)

[10] Afonso AS, Roque P, Fidelis L, Veras L, Conde A, Maranhão P, Leandro S, Hazin FH. 2020 Does lack of knowledge lead to misperceptions? Disentangling the factors modulating public knowledge about and perceptions toward sharks. Front. Mar. Sci. **7**, 663. (10.3389/fmars.2020.00663)

[11] McPhee D. 2014 Unprovoked shark bites: are they becoming more prevalent? Coast. Manag. **42**, 478-492. (10.1080/08920753.2014.942046)

[12] Ryan LA, Lynch SK, Harcourt R, Slip DJ, Peddemors V, Everett JD, Harrison L-M, Hart NS. 2019 Environmental predictive models for shark attacks in Australian waters. Mar. Ecol. Prog. Ser. **631**, 165-179. (10.3354/meps13138)

[13] West JG. 2011 Changing patterns of shark attacks in Australian waters. Mar. Freshw. Res. **62**, 744-754. (10.1071/MF10181)

[14] Tricas TC, McCosker JE. 1984 Predatory behavior of the white shark (*Carcharodon carcharias*), with notes on its biology. Proc. Calif. Acad. Sci. **43**, 221-238.

[15] Gruber S. 1988 Why do sharks attack people. Nav. Res. Rev. **40**, 2-19.

[16] Schultz LP. 1963 Attacks by sharks as related to the activities of man. In Sharks and survival. (eds PW Gilbert). Boston, MA: DC Heath & Co.

[17] Neff C, Hueter R. 2013 Science, policy, and the public discourse of shark ‘attack’: a proposal for reclassifying human–shark interactions. J. Environ. Stud. Sci. **3**, 65-73. (10.1007/s13412-013-0107-2)

[18] Johnson RH, Nelson DR. 1973 Agonistic display in the gray reef shark, *Carcharhinus menisorrah*, and its relationship to attacks on man. Copeia **1973**, 76-84. (10.2307/1442360)

[19] Semmens JM, Kock AA, Watanabe YY, Shepard CM, Berkenpas E, Stehfest KM, Barnett A, Payne NL. 2019 Preparing to launch: biologging reveals the dynamics of white shark breaching behaviour. Mar. Biol. **166**, 1-9. (10.1007/s00227-019-3542-0)

[20] Martin RA, Hammerschlag N, Collier RS, Fallows C. 2005 Predatory behaviour of white sharks (*Carcharodon carcharias*) at Seal Island, South Africa. J. Mar. Biol. Assoc. UK **85**, 1121-1135. (10.1017/S002531540501218X)

[21] Strong WR. 1996 Shape discrimination and visual predatory tactics in white sharks. In Great white sharks: the biology of Carcharodon carcharias (eds P Klimley, DG Ainley), pp. 229-240. San Diego, CA: Academic Press.

[22] Huveneers C, Holman D, Robbins R, Fox A, Endler JA, Taylor AH. 2015 White sharks exploit the sun during predatory approaches. Am. Nat. **185**, 562-570. (10.1086/680010) 25811089

[23] Litherland L. 2001 Retinal topography in elasmobranchs: interspecific and ontogenetic variations. Honours Thesis, Department of Anatomical Sciences, University of Queensland, Australia.

[24] Ritter E, Quester A. 2016 Do white shark bites on surfers reflect their attack strategies on pinnipeds? J. Mar. Biol. **2016**, 9539010. (10.1155/2016/9539010)

[25] Klimley AP, Anderson SD, Pyle P, Henderson R. 1992 Spatiotemporal patterns of white shark (*Carcharodon carcharias*) predation at the South Farallon Islands, California. Copeia **1992**, 680-690. (10.2307/1446143)

[26] Caldicott DG, Mahajani R, Kuhn M. 2001 The anatomy of a shark attack: a case report and review of the literature. Injury **32**, 445-453. (10.1016/S0020-1383(01)00041-9) 11476808

[27] Burgess G, Callahan M. 1996 Worldwide patterns of white shark attacks on humans. In The biology of Carcharodon carcharias (eds A Klimley, D Ainley), pp. 457-469. San Diego, CA: Academic Press.

[28] Hart NS, Theiss SM, Harahush BK, Collin SP. 2011 Microspectrophotometric evidence for cone monochromacy in sharks. Naturwissenschaften **98**, 193-201. (10.1007/s00114-010-0758-8) 21212930

[29] Schluessel V, Rick I, Plischke K. 2014 No rainbow for grey bamboo sharks: evidence for the absence of colour vision in sharks from behavioural discrimination experiments. J. Comp. Physiol. A **200**, 939-947. (10.1007/s00359-014-0940-0) 25245080

[30] Lisney TJ, Collin SP. 2008 Retinal ganglion cell distribution and spatial resolving power in elasmobranchs. Brain Behav. Evol. **72**, 59-77. (10.1159/000146082) 18679025

[31] Sokol S, Dobson V. 1976 Pattern reversal visually evoked potentials in infants. Investig. Ophthalmol. Vis. Sci. **15**, 58-62. 1245383

[32] Lisney TJ, Theiss SM, Collin SP, Hart NS. 2012 Vision in elasmobranchs and their relatives: 21st century advances. J. Fish Biol. **80**, 2024-2054. (10.1111/j.1095-8649.2012.03253.x) 22497415

[33] Ryan LA, Hart NS, Collin SP, Hemmi JM. 2016 Visual resolution and contrast sensitivity in two benthic sharks. J. Exp. Biol. **219**, 3971-3980. 27802139 10.1242/jeb.132100

[34] Ryan LA, Hemmi JM, Collin SP, Hart NS. 2017 Electrophysiological measures of temporal resolution, contrast sensitivity and spatial resolving power in sharks. J. Comp. Physiol. A **203**, 197-210. (10.1007/s00359-017-1154-z) 28247014

[35] Kalinoski M, Hirons A, Horodysky A, Brill R. 2014 Spectral sensitivity, luminous sensitivity, and temporal resolution of the visual systems in three sympatric temperate coastal shark species. J. Comp. Physiol. A **200**, 997-1013. (10.1007/s00359-014-0950-y) 25319537

[36] McComb DM, Frank TM, Hueter RE, Kajiura SM. 2010 Temporal resolution and spectral sensitivity of the visual system of three coastal shark species from different light environments. Physiol. Biochem. Zool. **83**, 299-307. (10.1086/648394) 20109067

[37] Ryan LA, Meeuwig JJ, Hemmi JM, Collin SP, Hart NS. 2015 It is not just size that matters: shark cruising speeds are species-specific. Mar. Biol. **162**, 1307-1318. (10.1007/s00227-015-2670-4)

[38] Marra NJ, Wang M, Sun Q, Pavinski Bitar PD, Stanhope MJ, Shivji MS. 2016 Mitochondrial genome of an Atlantic white shark (*Carcharodon carcharias*). Mitochondrial DNA B **1**, 717-719. (10.1080/23802359.2016.1222248) PMC7799651 33473604

[39] How MJ, Zanker JM. 2014 Motion camouflage induced by zebra stripes. Zoology **117**, 163-170. (10.1016/j.zool.2013.10.004) 24368147

[40] Pallus AC, Fleishman LJ, Castonguay PM. 2010 Modeling and measuring the visual detection of ecologically relevant motion by an *Anolis* lizard. J. Comp. Physiol. A **196**, 1-13. (10.1007/s00359-009-0487-7) 19908049

[41] Daniel MM, Alvermann L, Böök I, Schluessel V. 2021 Visual discrimination and resolution in freshwater stingrays (*Potamotrygon motoro*). J. Comp. Physiol. A **207**, 43-58. (10.1007/s00359-020-01454-2) PMC7875849 33263813

[42] Dowling JE, Ripps H. 1970 Visual adaptation in the retina of the skate. J. Gen. Physiol. **56**, 491-520. (10.1085/jgp.56.4.491) 5507093 PMC2225965

[43] Cohen JL. 1980 Functional organization of the retina of the lemon shark (Negaprion brevirostris, Poey): an anatomical and electrophysiological approach. Coral Gables, FL: University of Miami.

[44] Bates D, Mächler M, Bolker B, Walker S. 2015 Fitting linear mixed-effects models using lme4. J. Stat. Softw. 67, 1-48. (10.18637/jss.v067.i01)

[45] Zar JH. 1999 Biostatistical analysis, Englewood Cliffs, NJ: Prentice-Hall.

[46] Lenth R. 2016 Least-squares means: the R package lsmeans. J. Stat. Sotfware **69**, 1-33.

[47] Zhang D, Lu G. 2004 Review of shape representation and description techniques. Pattern Recognit. **37**, 1-19. (10.1016/j.patcog.2003.07.008)

[48] Yuan Z, Li F, Zhang P, Chen B. 2014 Description of shape characteristics through Fourier and wavelet analysis. Chin. J. Aeronaut. **27**, 160-168. (10.1016/j.cja.2013.07.011)

[49] Galletti C, Fattori P. 2003 Neuronal mechanisms for detection of motion in the field of view. Neuropsychologia **41**, 1717-1727. (10.1016/S0028-3932(03)00174-X) 14527536

[50] Andermann ML, Kerlin AM, Roumis DK, Glickfeld LL, Reid RC. 2011 Functional specialization of mouse higher visual cortical areas. Neuron **72**, 1025-1039. (10.1016/j.neuron.2011.11.013) 22196337 PMC3876958

[51] Goldman KJ, Anderson SD. 1999 Space utilization and swimming depth of white sharks, *Carcharodon carcharias*, at the South Farallon Islands, central California. Environ. Biol. Fishes **56**, 351-364. (10.1023/A:1007520931105)

[52] Hammerschlag N, Martin RA, Fallows C. 2006 Effects of environmental conditions on predator–prey interactions between white sharks (*Carcharodon carcharias*) and Cape fur seals (*Arctocephalus pusillus pusillus*) at Seal Island, South Africa. Environ. Biol. Fishes **76**, 341-350. (10.1007/s10641-006-9038-z)

[53] Hussey NE, McCann HM, Cliff G, Dudley SF, Wintner SP, Fisk AT. 2012 Size-based analysis of diet and trophic position of the white shark (*Carcharodon carcharias*) in South African waters. In Global perspectives on the biology and life history of the white shark (ed. M Domeier), pp. 27-49. Boca Raton, FL: CRC Press.

[54] Grainger R, Peddemors VM, Raubenheimer D, Machovsky-Capuska GE. 2020 Diet composition and nutritional niche breadth variability in juvenile white sharks (*Carcharodon carcharias*). Front. Mar. Sci. **7**, 422. (10.3389/fmars.2020.00422)

[55] Litherland L, Collin SP, Fritsches KA. 2009 Eye growth in sharks: ecological implications for changes in retinal topography and visual resolution. Vis. Neurosci. **26**, 397-409. (10.1017/S0952523809990150) 19698193

[56] Lisney TJ, Bennett MB, Collin SP. 2007 Volumetric analysis of sensory brain areas indicates ontogenetic shifts in the relative importance of sensory systems in elasmobranchs. Raffles Bull. Zool. **14**, 7-15.

[57] Hammerschlag N, Martin RA, Fallows C, Collier RS, Lawrence R. 2012 Investigatory behavior toward surface objects and nonconsumptive strikes on seabirds by white sharks, *Carcharodon carcharias*, at Seal Island, South Africa (1997–2010). In Global perspectives on the biology and life history of the white shark (ed. ML Domeier), pp. 91-103. Boca Raton, FL: CRC Press.

[58] Cliff G, Dudley S. 1991 Sharks caught in the protective gill nets off Natal, South Africa. 4. The bull shark *Carcharhinus leucas* valenciennes. S. Afr. J. Mar. Sci. **10**, 253-270. (10.2989/02577619109504636)

[59] Simpfendorfer CA, Goodreid AB, McAuley RB. 2001 Size, sex and geographic variation in the diet of the tiger shark, *Galeocerdo cuvier*, from Western Australian waters. Environ. Biol. Fishes **61**, 37-46. (10.1023/A:1011021710183)

[60] Ryan LA et al*.* 2021 A shark's eye view: testing the ‘mistaken identity theory’ behind shark bites on humans. *Figshare*. 10.1098/rsif.2021.0533 PMC8548079 34699727

